# Finding sRNA generative locales from high-throughput sequencing data with NiBLS

**DOI:** 10.1186/1471-2105-11-93

**Published:** 2010-02-18

**Authors:** Daniel MacLean, Vincent Moulton, David J Studholme

**Affiliations:** 1 The Sainsbury Laboratory, John Innes Centre, Colney Lane, Norwich, NR4 7UH, UK; 2 University of East Anglia, Norwich, NR4 7TJ, UK

## Abstract

**Background:**

Next-generation sequencing technologies allow researchers to obtain millions of sequence reads in a single experiment. One important use of the technology is the sequencing of small non-coding regulatory RNAs and the identification of the genomic locales from which they originate. Currently, there is a paucity of methods for finding small RNA generative locales.

**Results:**

We describe and implement an algorithm that can determine small RNA generative locales from high-throughput sequencing data. The algorithm creates a network, or graph, of the small RNAs by creating links between them depending on their proximity on the target genome. For each of the sub-networks in the resulting graph the clustering coefficient, a measure of the interconnectedness of the subnetwork, is used to identify the generative locales. We test the algorithm over a wide range of parameters using RFAM sequences as positive controls and demonstrate that the algorithm has good sensitivity and specificity in a range of *Arabidopsis *and mouse small RNA sequence sets and that the locales it generates are robust to differences in the choice of parameters.

**Conclusions:**

NiBLS is a fast, reliable and sensitive method for determining small RNA locales in high-throughput sequence data that is generally applicable to all classes of small RNA.

## Background

High-throughput sequencing technologies such as Illumina's Solexa, 454 Life Sciences' GS-FLX and ABI's SOLiD platforms allow researchers to generate gigabases of sequence data in a matter of hours [1]. As such they are finding use in the analysis of many biological datasets, including the deep sequencing and cataloguing of non-coding small regulatory RNAs (sRNAs). These sRNAs have been described as the 'dark matter of genetics' [2] because they are highly abundant yet difficult to detect. They have roles in regulating gene expression via post-transcriptional and translational mechanisms in animals, fungi and plants. Single-stranded silencing RNAs of 21-25 nt in length, are created from a double stranded RNA by the protein Dicer. The RNAs are the guide for AGO nucleases that cleave the targeted RNA in a sequence specific manner. Cleaved RNAs are degraded further or become template for RNA-dependent polymerase to generate a dsRNA [3,4]. The known number of classes of sRNAs is great and with the advent of high-throughput sequencing is getting greater. With these recent advances in sequencing technology we are in a position to find new classes of sRNA that have not previously been discovered. The first step in this is in the identification of parts of the genome that generate sRNAs. We call these regions "locales", choosing this word for the obvious similarity to the term locus from the genetic literature, which defines a distinct point or region on a genome. It is the detection of locales with which this paper is concerned. After generating the sequence the reads must be aligned to the genome. Alignment is a well studied problem and is handled by a range of programs such as SSAHA [5], MAQ [6] and SOAP [7] (see [1] for a review and other alternatives). Grouping the reads into locales that represent the place of origin of potential functional sRNAs is the next step.

There has been little discussion of what constitutes a sRNA-generating locale, with researchers sometimes relying on restrictive and arbitrary definitions [8-10]. Many existing tools rely on the detection of specific classes of sRNA. For example, mirCat [11] and mirDeep [12] are micro-RNA (miRNA) detectors. Chen *et al*. have created a tool for predicting trans-acting siRNA (ta-siRNA) [13]. Other studies have used time-series data-mining algorithms to identify genomic locales from which sRNAs originate with disregard to sRNA class [14], but to date have relied on identifying only those that were statistically more 'unusual' than others according to their own measures. Such a method is not necessarily useful as it would lack the sensitivity to find the majority of locales. To avoid these problems, researchers have previously used simple but functional tools for generative region detection [11]. Thus there is a need for generally applicable, sensitive methods for determining locales from sequencing data. Since the full range of different classes of sRNA is not yet known search strategies for potential functional locales must be general.

**Figure 1:**
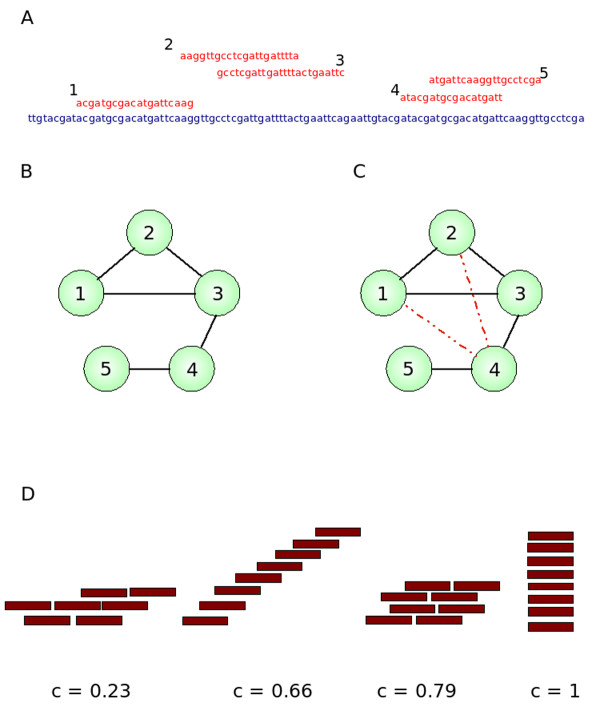
**Creation of a graph and calculation of clustering coefficient from sRNA sequence data**. A) sRNAs 1 - 5 are aligned to the target genome. B) The graph is then created, each of the green circles is a vertex that represents a sRNA and an edge (black line) is drawn between them if the sRNAs are close enough to each other on the genome. Each interconnected vertex-island is called a component and, for simplicity a single vertex island is shown. C) For each vertex in each component in the graph, the clustering coefficient is calculated, ie the ratio of the number of edges that are found between neighbours of the vertex (black lines) to the number of edges that could exist between them (red lines are edges that could exist, but do not). For example, vertex 1 connects to vertex 2 and 3. Just one edge could exist between 2 and 3, and one edge does exist, so the clustering coefficient for this node is 1/1, or 1. Similarly, vertex 3 has edges to vertices 1, 2 and 4. Three edges could exist between these three vertices but only one does (between 1 and 2), thus the clustering coefficient for vertex 3 is 1/3. The clustering coefficient of the entire component is the average of the individual clustering coefficients for each node. D) Example patterns of overlap and their corresponding clustering coefficients (c).

In this paper we propose and test a locale detection algorithm that we call *NiBLS *(for Network Based Locale Search) which takes a graph-theoretic approach to identifying locales. A graph is a mathematical abstraction that is particularly suited to the description of relationships between entities (see [15] for a discussion). Here a graph consists of vertices and edges that are links between the vertices. In our graphs the vertices are the sRNAs and the edges link sRNAs on the basis of proximity (Figure 1A and 1B). We use proximity within an absolute cut-off to create edges between the sRNA vertices. Once the edge is created the information about the distance is discarded. Many graphs are composed of isolated vertex-islands, termed components, that have edges between vertices within themselves, but not with other vertex-islands. The clustering coefficient [16] of a component is a measure of the degree of inter-connectivity within it (Figure 1C). Each vertex has a certain number of neighbours, and the clustering coefficient is a function of the number of edges between the neighbours and the maximum possible number of edges between them and high levels of interconnectivity equate to large clustering coefficients (Figure 1D). Our algorithm uses clustering coefficients in the graph of sRNAs to detect locales as individual highly clustered components, not as it may seem at first glance the density of sRNAs on the reference.

## Results and Discussion

### Algorithm

#### Definition and detection of locales

A locale is defined as a component of a graph *G *= (*V, E*) with vertices *V *and edges *E *that has clustering coefficient *γ *above a user-defined cutoff *C*. To create the graph we align sRNAs to the target genome such that *s *is a sRNA on chromosome *c *with start *i *and end *j*.

The vertices of *G *are the set of sRNAs,(1)

An edge *e *exists between two sRNAs if the overlap (or distance between) is less than the minimum inclusion distance *M*, that is(2)

is an edge if(3)

For each connected set of sRNAs (i.e. each component *l *of *G*) the clustering coefficient *γ *as defined by Watts and Strogatz [16] is the average of the ratio of the number of edges that exist between the neighbours of each vertex in the component and the number that could possibly exist. The final set of locales *L *comprises all components with more than one sRNA and *γ *> *C*. That is,(4)

The extent of each locale is from the lowest start (*i*) to the highest end (*j*) for each sRNA in the component *l*.

### Testing

#### Sensitivity and specificity of the algorithm

To test whether our algorithm is capable of detecting biologically meaningful locales from sRNA data, we examined its sensitivity and specificity on publicly available high-throughput sRNA pyrosequencing of sRNAs extracted from the flowers, rosettes or entire seedlings of the higher plant *Arabidopsis thaliana *[8] and mouse embryonic stem (ES) cells [17]. Typically, sensitivity of an algorithm is assessed by comparison of some output against a pre-known result. However, there is no organism or tissue in which the full set of expressed sRNA and generative locales is known; thus it is difficult to establish a comprehensive set of true positive locales for comparison.

To address this issue the set of RFAM sequences [18] known for each species (excluding RFAM sequences for rRNAs and tRNAs) was considered to be the positive control set of sRNAs against which the putative locales generated by our algorithm would be tested. By its nature this is a somewhat problematic control standard; the RFAM database does not comprehensively include all sRNAs and not all RFAM RNAs are expressed in all tissues. This means our algorithm could detect true positive locales that do not match RFAM sequences, thereby appearing to be a false positive. Conversely an ncRNA may not be expressed in the tissue of interest leading to a true negative that appears to be a false negative. We therefore excluded each RFAM sequence that had fewer than 5 genomic matches aligned to it. As such, all 'real' locales under consideration stood a chance of being detected from the data. After filtering, the number of RFAMs remaining as potential positive control locales in each species was considerably reduced from the total possible (Table 1). However, there was a large number of nucleotides to which sRNAs could be aligned allowing for a reasonable assessment of the number of nucleotides grouped into putative locales.

**Table 1:** Number of RFAMs in each tissue.

Species	Total number of RFAMs	Tissue	RFAMs > 5 hits	nt
*Arabidopsis*	84	Flower	22	3686

-	-	Rosette	18	2850

-	-	Seedling	37	5638

Mouse	492	Embryonic Stem Cells	16	2237

Table describes the total number of RFAMs for each species, the number of RFAMs with more than 5 sRNAs that align to them in each tissue and the total number of nt that these comprise.

**Figure 2:**
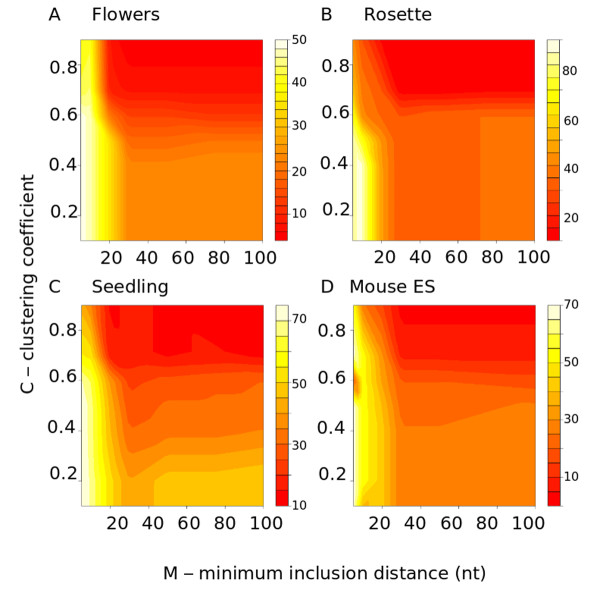
**Sensitivity of the algorithm for various values of C and *M***. Heatmaps showing the sensitivity of the algorithm in detecting RFAM locales from sRNA sequence sets derived from different tissues in *Arabidopsis thaliana*. For each value of the parameters *C *- the clustering coefficient and *M *- the minimum inclusion distance, the sensitivity of the algorithm was calculated. *x *axis = minimum inclusion distance in nt, *y *axis = clustering coefficient. Colour scale indicates the degree of sensitivity for the tissue. A) sensitivity analysis on sRNAs sequenced from flowers, B) from rosette tissue, C) from seedling tissue and D) from mouse ES cell.

We tested our algorithm at a range of values of the two parameters: *M *the minimum inclusion distance in nucleotides at which an edge is created between them and *C *the minimum clustering coefficient at which a component in the graph is deemed a locale. The sensitivity and specificity of the algorithm were calculated as described in Methods. Exploratory runs with *Arabidopsis *and mouse data showed that results changed little for values of *M *over 100, so scan values were kept below this threshold (Additional Files 1, 2, 3, 4). The sensitivity of the algorithm in detecting RFAM locales expressed in different sets of sRNA sequenced from different tissues of *Arabidopsis *can be seen in Figure 2. Generally sensitivities, which could possibly fall in the range 0 to 100, are good, with the maximum sensitivities in each parameter scan ranging from 75.85 to 48.93, indicating that the algorithm has good detection capability. In all the *Arabidopsis *and mouse tissues tested here the algorithm had greatest sensitivity at low *M*. For *M *< 20 the highest sensitivities were 75.85 in the rosette, 74.7 for the seedling tissue, 48.93 in the flower and 69.21 in mouse ES (Figure 2A-D). Sensitivity is much lower at *M *> 20 with sensitivities dropping off sharply in flowers and rosette tissues, although somewhat less so in the seedling tissue and mouse ES cells. Together these results suggest that the *M *parameter, the minimum inclusion distance, is the most important factor in the algorithm's ability to discern locales. However, the parameter *C *has an important modulating role and can become substantially limiting on sensitivity as it increases, especially at *M *> 20. In the *M *< 20 region of greatest sensitivity the exact point at which *C *becomes limiting is different in each tissue but generally when *C *> 0.6 sensitivity is less than 40. A sharp cutoff is seen in the rosette and flower tissue (Figure 2A and 2B) and a more gradual one in the seedling and mouse (Figure 2C and 2D). Interestingly the sensitivity increases slightly for *M *> 40 in seedlings and to a lesser extent in rosette (Figure 2B). This may be due to the occasional appearance in the sequence set of low-abundance sRNAs that align to regions of genome that when transcribed are found on the complementary strand of a hairpin structure.

The *Caenorhabditis elegans *sRNA complement includes a huge number of well known and well annotated sRNAs, such as the 21U-RNAs, a class of RNAs whose sequence begins with uracil and have length of 21 nt [19]. It could be argued that this provides an excellent test case as many of the real locales are known. However, the know loci in this case are very easy to detect, having specific mapping points on the reference genome. We added 21U-RNA to our sample and carried out the analysis as described above in *C.elegans*. The sensitivity of the algorithm in this case was very high (Additional File 5) and never drops to be as low as that in the other tests. At 75% of parameter values we used over 40% of loci are recovered. In this case we believe that the large number of 21U-RNAs (>15000) [19] is skewing the result and giving a perhaps non-representative view of the efficacy of the algorithm for general use.

The specificity of the algorithm was high: greater than 90 in all tissues at all parameters (see Additional Files 6, 7). In part this is because it is not possible for the algorithm to detect locales where there are no sRNAs aligned and so it cannot spontaneously generate false positives. Furthermore, for a locale to exist the definition requires that a component *l *of the graph should have at least two vertices. This removes all sRNAs separated by more than *M *from others, since, in redundant sequence sets, the real locales would be expected to be represented by more than one sequence. Such a factor has the effect of greatly reducing the 'junk' that could be considered for inclusion in locales. Together these results show clearly that the algorithm can sensitively and specifically identify sRNA locales in sRNA sequence data from evolutionarily distantly related species. In the *Arabidopsis *and mouse sequence data tested here it seems that parameter settings for optimal sensitivity fall in the range 0 <*M *< 20 and 0 <*C *< 0.6.

It is important to note the necessary differences in interpretation of the value of the clustering coefficient in the context of co-overlapping sRNAs and the interpretation used in the network literature, in particular the primary article of Watts and Strogatz [16]. Graphs created by randomly assigning edges between nodes typically have a lower clustering coefficient than real-world networks, biological networks such as the *Caenorhabditis elegans *neuronal network have clustering coefficients on the order of 0.3, random networks of around 0.05 [16]. The high clustering coefficient implies that the nodes in the real-world networks share many neighbours with their neighbours and suggests the structure of the network is modular. In our algorithm we use the clustering coefficient simply as a measure of the co-overlapping of the sRNAs and if we find a sufficiently high co-overlapping pattern we have a candidate locale. The effective values are in the range 0 <*C *< 0.6 which shows that the reads from sequencing experiments and different types of sRNA co-overlap in a wide variety of patterns, thus the clustering co-efficient reflects the structure of the potential locale. Locales in which the sRNA reads overlap in a serial manner on the reference one after the other in a 'fallen domino' sort of pattern will have lower clustering coefficients, whereas locales in which sRNA reads are piled high on the reference, each overlapping many other sRNA reads more akin to the bricks in a wall will have higher clustering coefficients. The exact value of the clustering coefficient cut-off could conceivably be manipulated to narrow ranges to find locales with specific sRNA alignment patterns, although in this paper the aim is to retain as wide a selection as possible.

#### Reproducibility of results at different parameter settings

**Figure 3:**
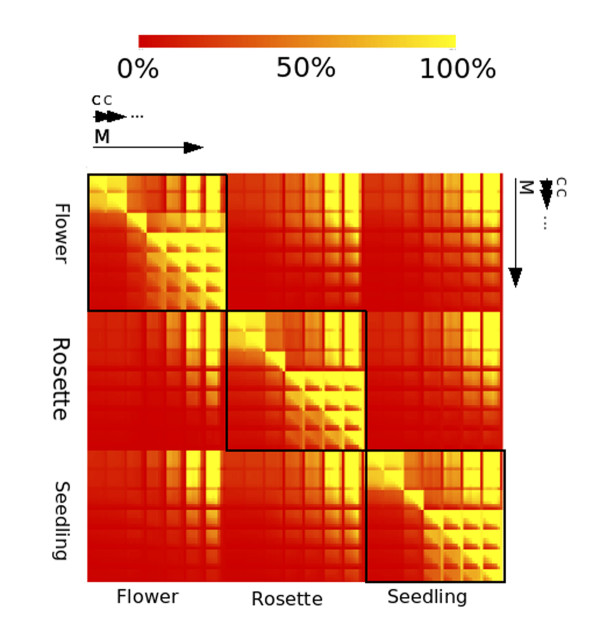
**Pairwise comparisons of overlap of sRNA locales generated at all parameter scan values for all sets of Arabidopsis tissues**. Within each of the nine visible sub-squares all *M *values (5, 10, 20, 30, 50, 75, and 100) occur once and all *C *values occur once for each *M *repeating a total of seven times within each sub-square. The extent of one scale of *M *is indicated by one large arrow, the extent of one scale of *C *is indicated by one small arrow. For each comparison the proportion of overlapping locales is calculated as the number of locales in the locales set represented on the *x *axis that overlap with the locales in the set represented on the *y *axis.

In order to assess the extent to which the algorithm could generate similar results from different parameter settings for each tissue we examined the overlap on the reference genome of the sets of locales generated by the algorithm for all values of *M *and *C *used in the parameter scans. Locale sets were examined in a pairwise fashion and the proportion of locales with an overlap in genomic position with a locale in the corresponding set calculated. In a situation where the total number of locales in set A is different to the total number of locales in set B the percentage of locales present in both will vary depending on which set you consider to be the reference set. Consider set A contains 50 locales and set B contains 100 locales. If set B is used as a reference set and all 50 of set A are present in set B we will have found 50% of our reference locales. Conversely if we use set A as the reference set we will find 100% of our reference locales. Rather than causing a discrepancy in the analysis, this difference can tell us about the relative numbers of locales generated by different settings, so in our pairwise comparisons we used each locales set as the reference set in turn. Differences in proportion of genomic position overlapping locales caused by different numbers of locales are easily identified as asymetrical regions about the top-left to bottom-right diagonal in Figure 3. Similar parameter values generate very similar sets of locales; this is seen as the bright yellow area around the top left to bottom right diagonal in Figure 3A. The algorithm shows the same reproducibility characteristics in the three different *Arabidopsis *sRNA sets. The pattern is repeated in each of the large outlined boxes along the diagonal in Figure 3A indicating that the characteristics of reproducibility are the same in each tissue. Within each tissue, close parameter values generate very similar sets of locales. This is seen as the bright yellow colour around the top left to bottom right diagonal in each box. For *M *< 10, reproducibilty is high then drops when 30 <*M *< 75 and increases again when *M *> 75, possibly reflecting the inclusion of multiple smaller locales into larger ones by virtue of the increasing *M*, the minimum inclusion distance. As *M *increases some locales with relatively small distances can be merged into one another. For *M *> 20, the reproducibility is high but there are differences in the number of locales in each set, visible as differences in colour above and below the diagonal in the bottom-right area of each square in Figure 3A. This may be a consequence of an increased inclusion distance merging locales that are separate in one set. The number of locales in each set is similar where *M *< 20, reproducibility remains high in this range, visible as similar colour above and below the diagonal in the top-left of each box.

**Figure 4:**
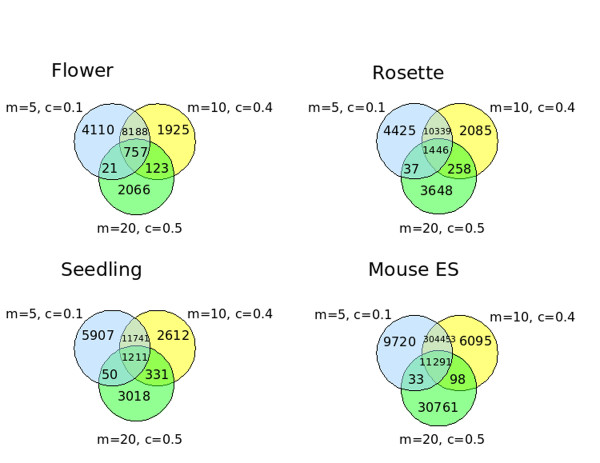
**Venn diagrams of numbers of exactly the same locales appearing in Arabidopsis and mouse tissues at 3 sets of paramater values within the sensitive range**. The number of locales with exactly the same start and stop coordinates on the same chromosomes appearing uniquely in each parameter set or in all combinations of sets were calculated.

To give an impression of the number of exactly identical locales that were generated at different parameter values we selected three pairs of values for *M *and *C *(*M *= 5, 10, 20, *C *= 0.1, 0.4, 0.5) that were in the sensitive and reproducible range of parameter values for both *Arabidopsis *and mouse and calculated the number of locales with the same exact start and stop positions. The Venn diagrams in Figure 4 show that the proportion of shared identical locales varies from 5.78% to 26.83%. Although each set had a large number of unique locales these must overlap at least one other locale on the genome in the corresponding set since there is high reproducibility over the same range. The number of shared identical locales was much higher between sets from close parameter values than the divergent ones. Overall, the high reproducibility for similar parameter values across the range and the general decrease in number of locales shared as the parameter values diverge indicates that the algorithm is robust to moderate differences in parameter value.

#### Genomic features with sRNA locales

**Figure 5:**
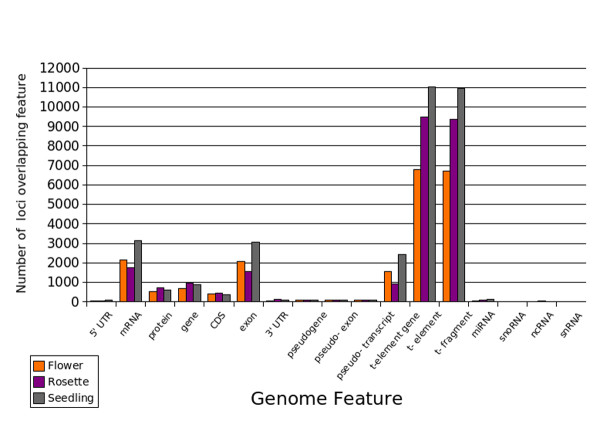
**Arabidopsis genomic features overlapped by locales generated with parameter values *M *= 5 and *C *= 0.25**. TAIR 8 genome annotations were used as reference features and the number of locales overlapping each genomic feature was calculated. All nested features e.g genes within transposable elements were marked with overlaps equally as appropriate.

We counted the number of locales that overlapped different classes of genomic feature in *Arabidopsis*. For this analysis we used a set of locales generated with *M *= 5, *C *= 0.25. The genomic feature types most mapped over are the transposon related elements, transposons, transposable element genes and transposable fragments (Figure 5). Although not many sRNA features are annotated in *Arabidopsis *locales mapping to miRNA, snoRNA, ncRNA and snRNA were found in all tissues. For example in flower, rosette and seedling tissue 63, 81 and 129 locales mapped to the 176 annotated miRNAs. mRNAs and exon features were also relatively well mapped over by locales, though the proportion of the total number of these elements mapped over was lower than the proportion of the transposon-related elements.

### Implementation

#### Standalone Perl version

Our algorithm has been implemented in Perl [20] to provide an easy to run multi-platform package that can be incorporated easily into analysis pipelines. This implementation is limited only by local system resources. To gain optimal performance from graph analyses which can be computationally expensive, we have used the Boost Graph Library [21], implemented in C++ and available free to academic users under the Boost Graph License and the Perl interface Boost-Graph module [22], available under the GNU public license [23]. Both of these pieces of software are pre-requisites for running the implementation. Our implementation is released under GPL3 [23]. The Perl implementation requires as input a GFF format file [24] describing the alignment of sRNAs to the reference genome. As guide to performance, with the 213,799 mapped sRNAs in the *Arabidopsis *flower data [8], our Perl implementation ran in 37 minutes on an AMD64 IBM Intellistation Desktop with 2 Gb of RAM.The Perl implementation can be obtained from github [25].

## Conclusions

We have created an algorithm that uses a graph theoretical approach to identify sRNA generative locales from high-throughput sequencing data. Despite the huge evolutionary distance between *Arabidopsis *and mouse the algorithm was capable of correctly identifying locales with very high sensitivity and with similar patterns of sensitivity for both of the species, suggesting that it has applicability across the plant and animal kingdoms. The sets of locales generated by the algorithm's user-definable parameters *M *and *C *are robust to small changes over the possible range whereas larger differences have greater effects indicating that the algorithm is both robust and responsive. With our stand-alone Perl implementation it is possible for a user carry out a parameter scan at the start of an analysis to identify the parameter values of greatest sensitivity and specificity for their sequence set if necessary.

One difficulty all sRNA locus finding algorithms must deal with is the fact that not all sRNAs from high-throughput sequencing experiments will be 'functional' and depending on the sequencing protocol used many of the sRNAs could be a result of degradation processes which a researcher may not have interest in. The literature does not yet contain a consensus on what such a degradation locus may look like, making it difficult for algorithms to distinguish such locales from those of functional interest in any generally useful way at present. Nonetheless in such situations our algorithm can be of use in filtering out potential non-functional locales in cases where the researcher has prior expectation of the pattern formed by degradation products. For example in the case where degradation products have a distinctive visual pattern, representative locales matching the pattern can be identified visually in a genome browser and comparing an initial run of the algorithm with positions of the pattern. The clustering coefficients of the locales can then be used as a band-filter whereby any locales lower or higher than this can be presumed not to be from the same sort of degradation process.

As our algorithm uses only positional data of aligned sRNAs and the clustering coefficient cut-off to identify locales it is naturally sRNA class agnostic which mean it can be used to identify locales of many different kinds at once as well as, potentially, previously unknown classes of locales. Typically the number of locales called is many times greater than the number of locales known as RFAMs for a given species, for example in the *M *= 10, *C *= 0.4 set discussed in Figure 4 10,000 locales are predicted. This indicates that there are a huge number of sRNA generative locales and sRNAs not yet known, fully justifying the description of them as the dark matter of genetics. Undoubtedly there is much scope for many different methods for detection of sRNA locales. Furthermore, the identification and cataloguing of sRNA generative locales could help the development of methods that can predict generative locales *de novo *from genomic sequence.

## Methods

### Alignment of sequences to reference genomes

Publicly available data from small RNA deep sequencing experiments were downloaded from the Gene Expression Omnibus [26] with accession numbers GSM118373 (*Arabidopsis thaliana*) [8] and GSM314558 (*Mus musculus*) [17]. RFAMs and sequences for each species were obtained from RFAM [18]. Sequences were aligned to either the TAIR 8 [27]*Arabidopsis *sequence or the mm9 mouse assembly build 37 hosted at UCSC [28], using SSAHA 3.1 [5]. For sRNA alignment redundant sequence sets were used and only sequences matching to the reference with 100% identity over 100% of the sequence length were retained. Sequences aligning to more than one position on the reference genome were not removed or normalised in any way, meaning a sRNA that belongs to one position may appear as if it comes from many. Parsing and collation was done with custom Perl scripts.

### Parameter Scans

To systematically determine the sensitivity and specificity of the algorithm, we carried out 'parameter scans', a series of runs of the algorithm on each dataset changing the value of one of the paramaters at each run. The *M *parameter (minimum inclusion distance) was tested at values of 5, 10, 20, 30, 50, 75, and 100. Early runs with the *Arabidopsis *data showed that results changed little when *M *values exceeded 100. Values of *C *were 0.1, 0.25, 0.4, 0.5, 0.6, 0.75 and 0.9.

### Calculation of Sensitivity and Specificity

For sensitivity and specificity analyses, the number of true positives (*TP*) was calculated as the number of nucleotides in the genome with an RFAM alignment and a putative locale alignment. True negatives (*TN*) were calculated as the number of nucleotides in the reference genome with neither a filtered RFAM alignment nor a putative locale alignment. False positives (*FP*) were calculated as a nucleotide in the genome that aligned to a putative locale but had no RFAM aligned. False negatives (*FN*) were calculated as nucleotides in the genome with no putative locale aligned and an RFAM aligned.

Sensitivity was calculated as:(5)

Specificity was calculated as:(6)

### Overlapping elements

For calculation of numbers of overlapping genomic features in different locales sets and relative to genome annotations Perl scripts were used. Reference annotations were obtained as GFF from TAIR [27].

### Visualisation of Results

Contour graphs were created by using the R package *akima *[29] to carry out bivariate interpolation of the irregularly spaced parameter scan data onto a regularly spaced grid with the *interp *and *filled.contour *functions. Heatmaps were generated using MeV 4 [30]

## Availability and Requirements

Project name: NiBLS

Project home page: http://github.com/danmaclean/NiBLS

Operating system(s): Platform independent

Programming language: Perl

Other requirements: Perl 5.6 or higher, Perl Boost::Graph module, also under GPL and available from http://search.cpan.org/~dburdick/Boost-Graph-1.2/Graph.pm

License: GPL 3

Restrictions to use by non-academics: none

## Authors' contributions

DM conceived of the locale identification method, created the implementation, conceived of and carried out the tests and co-wrote the paper. DJS conceived of the tests and co-wrote the paper and VM co-wrote the paper. All authors have read and approved the manuscript.

## Supplementary Material

Additional file 1 Parameter scans for *M *> 100 in sRNA from *Arabidopsis thaliana* Flower. Click here for file

Additional file 2 Parameter scans for *M *> 100 in sRNA from *Arabidopsis thaliana* Rosette. Click here for file

Additional file 3 Parameter scans for *M *> 100 in sRNA from *Arabidopsis thaliana* Seedling. Click here for file

Additional file 4 Parameter scans for *M *> 100 in sRNA from mouse ES cells. Click here for file

Additional file 5 Parameter scans from sRNAs from *C. elegans*. Click here for file

Additional file 6 Summary of parameter scans for sensitivity and specificity in mouse ES cells. Click here for file

Additional file 7 Summary of parameter scans for sensitivity and specificity in *Arabidopsis thaliana*. Click here for file

## References

[B1] MacLean D Jones JDG Studholme DJ Application of 'Next Generation' sequencing technologies to microbial genetics Nat Revs Microbiol 2009 7 4 287 296 10.1038/nrmicro2122 19287448

[B2] Baulcombe DC RNA silencing in plants Nature 2004 431 356 363 10.1038/nature02874 15372043

[B3] Brodersen P Voinnet O The diversity of RNA silencing pathways in plants Trends Genet 2006 22 268 280 10.1016/j.tig.2006.03.003 16567016

[B4] Lippman Z Martienssen R The role of RNA interference in heterochromatic silencing Nature 2004 431 364 370 10.1038/nature02875 15372044

[B5] Ning Z Cox AJ Mullikin JC SSAHA: a fast search method for large DNA databases Genome Res 2001 11 1725 1729 10.1101/gr.194201 11591649 PMC311141

[B6] Li H Ruan J Durbin R Mapping short DNA sequencing reads and calling variants using mapping quality scores Genome Res 2008 18 11 1851 1858 10.1101/gr.078212.108 18714091 PMC2577856

[B7] Li R Li Y Kristiansen K Wang J SOAP: short oligonucleotide alignment program Bioinformatics 2008 24 5 713 714 10.1093/bioinformatics/btn025 18227114

[B8] Rajagopalan R Vaucheret H Trejo J Bartel DP A diverse and evolutionarily fluid set of microRNAs in *Arabidopsis thaliana* Genes Dev 2006 20 3407 3425 10.1101/gad.1476406 17182867 PMC1698448

[B9] Molnar A Schwach F Studholme DJ Thuenemann EC Baulcombe DC miRNAs control gene expression in the single-cell alga *Chlamydomonas reinhardtii* Nature 2007 447 1126 1129 10.1038/nature05903 17538623

[B10] Mosher RA Schwach F Studholme D Baulcombe DC PolIVb influences RNA-directed DNA methylation independently of its role in siRNA biogenesis Proc Nat Acad Sci USA 2008 105 3145 3150 10.1073/pnas.0709632105 18287047 PMC2268599

[B11] Moxon S Schwach F Dalmay T MacLean D Studholme DJ Moulton V A toolkit for the analysis of large-scale plant small RNA datasets Bioinformatics 2008 24 19 2252 2253 10.1093/bioinformatics/btn428 18713789

[B12] FriedlÃnder MR Chen W Adamidi C Maaskola J Einspanier R Knespel S Rajewsky N Discovering microRNAs from deep sequencing data using miRDeep Nat Biotechnol 2008 26 407 415 10.1038/nbt1394 18392026

[B13] Chen HM Li YH Wu SH Bioinformatic prediction and experimental validation of a microRNA-directed tandem trans-acting siRNA cascade in *Arabidopsis* Proc Natl Acad Sci USA 2007 104 3318 3323 10.1073/pnas.0611119104 17360645 PMC1805617

[B14] Bagnall AJ Moxon S Studholme D Time-series data-mining algorithms for identifying short RNA Arabidopsis thaliana, UEA Technical Report CMP-C07-02" 2008

[B15] Huber W Carey VJ Long L Falcon S Gentleman R Graphs in molecular biology BMC Bioinformatics 2007 8 S8 10.1186/1471-2105-8-S6-S8 PMC1995545 17903289

[B16] Watts DJ Strogatz SH Collective dynamics of 'small-world' networks Nature 1998 393 409 410 10.1038/30918 9623998

[B17] Babiarz JE Ruby JG Wang Y Bartel DP Blelloch R Mouse ES cells express endogenous shRNAs, siRNAs, and other Microprocessor-independent, Dicer-dependent small RNAs Genes Dev 2008 22 2773 2785 10.1101/gad.1705308 18923076 PMC2569885

[B18] RFAM http://rfam.sanger.ac.uk/

[B19] Ruby JG Jan C Player C Axtell MJ Lee W Nusbaum C Ge H Bartel DP Large-scale sequencing reveals 21U-RNAs and additional microRNAs and endogenous siRNAs in C. elegans Cell 2006 127 6 1193 1207 10.1016/j.cell.2006.10.040 17174894

[B20] The Perl Directory http://www.perl.org

[B21] Boost Graph Library http://www.boost.org/

[B22] Boost-Graph-1.2 Perl module http://search.cpan.org/~dburdick/Boost-Graph-1.2/Graph.pm

[B23] The GNU Public License Version 3 http://www.gnu.org/licenses/gpl-3.0.txt

[B24] GFF file format http://www.sanger.ac.uk/Software/formats/GFF/

[B25] The Sainsbury Laboratory ncRNA webserver http://github.com/danmaclean/NiBLS

[B26] The Gene Expression Omnibus http://www.ncbi.nlm.nih.gov/geo/

[B27] The Arabidopsis Information Resource http://arabidopsis.org

[B28] The UCSC Genome Bioinformatics Website http://hgdownload.cse.ucsc.edu/goldenPath/mm9/chromosomes/

[B29] CRAN - Comprehensive R Archive Network, akima package http://cran.r-project.org/web/packages/akima/index.html

[B30] Dudoit S Gentleman RC Quackenbush J Open source software for the analysis of microarray data Biotechniques 2003 Suppl 45 51 12664684

