# Supplementary figures and images for: Finding sRNA generative locales from high-throughput sequencing data with NiBLS
Source: BMC Bioinformatics. 2010 Feb 18;11:93. doi: 10.1186/1471-2105-11-93 (PMC2837031; doi:10.1186/1471-2105-11-93)

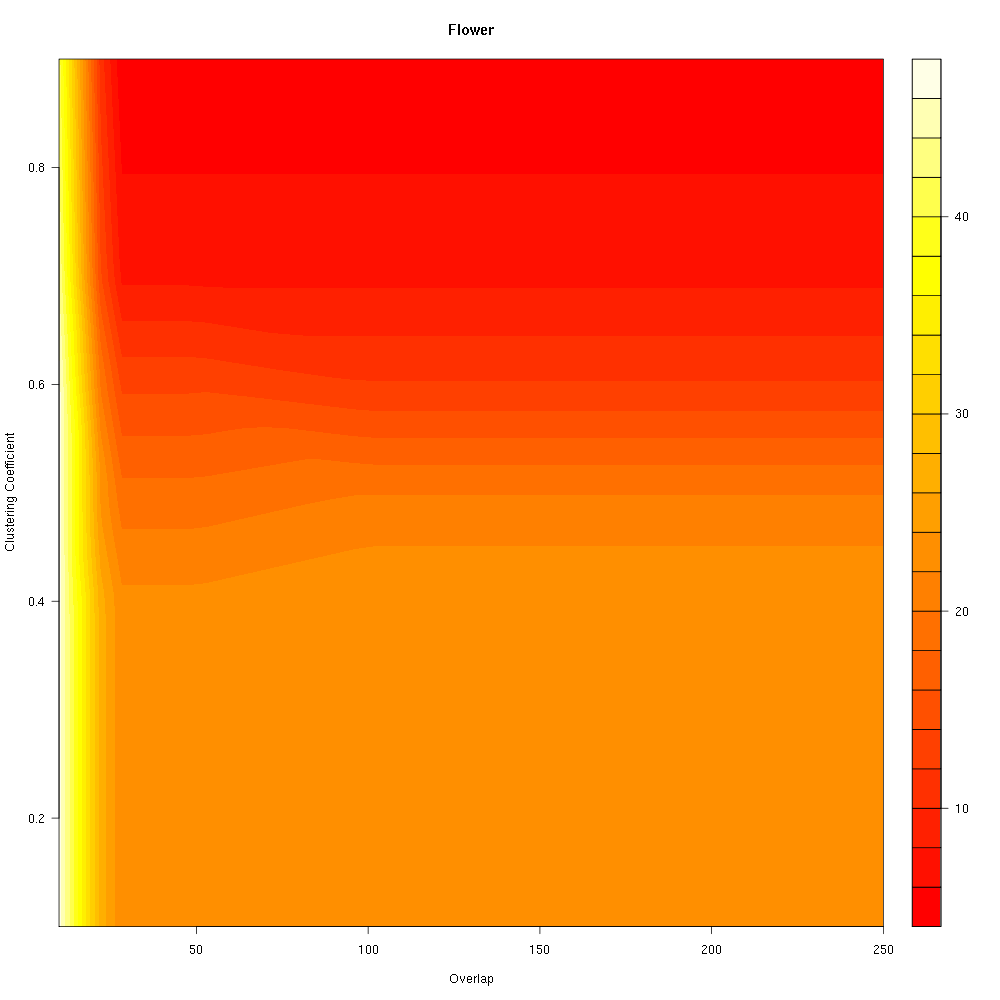

Supplement: Additional file 3 — Parameter scans for M > 100 in sRNA from Arabidopsis thaliana Seedling. [file 1471-2105-11-93-S3.PNG]

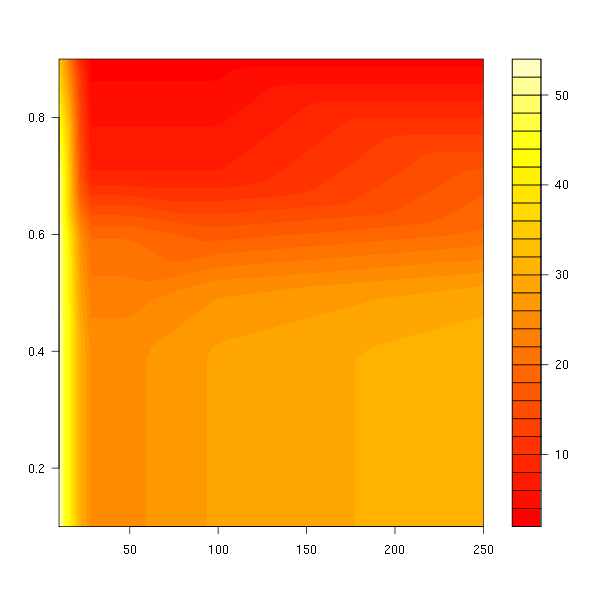

Supplement: Additional file 4 — Parameter scans for M > 100 in sRNA from mouse ES cells. [file 1471-2105-11-93-S4.PNG]

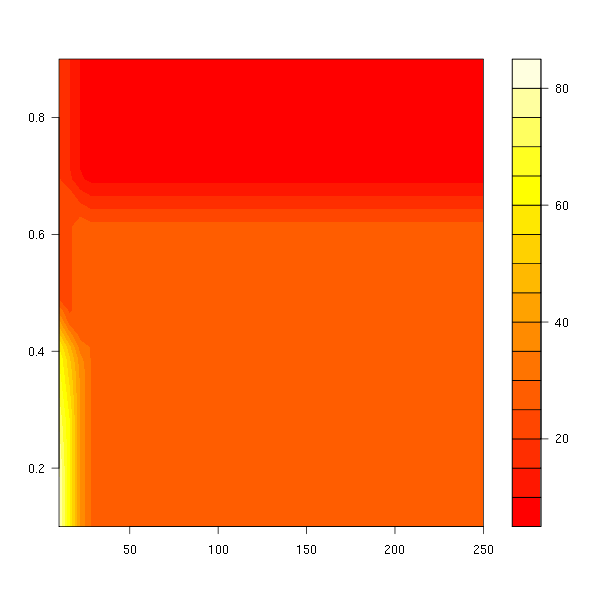

Supplement: Additional file 5 — Parameter scans from sRNAs from C. elegans. [file 1471-2105-11-93-S5.PNG]

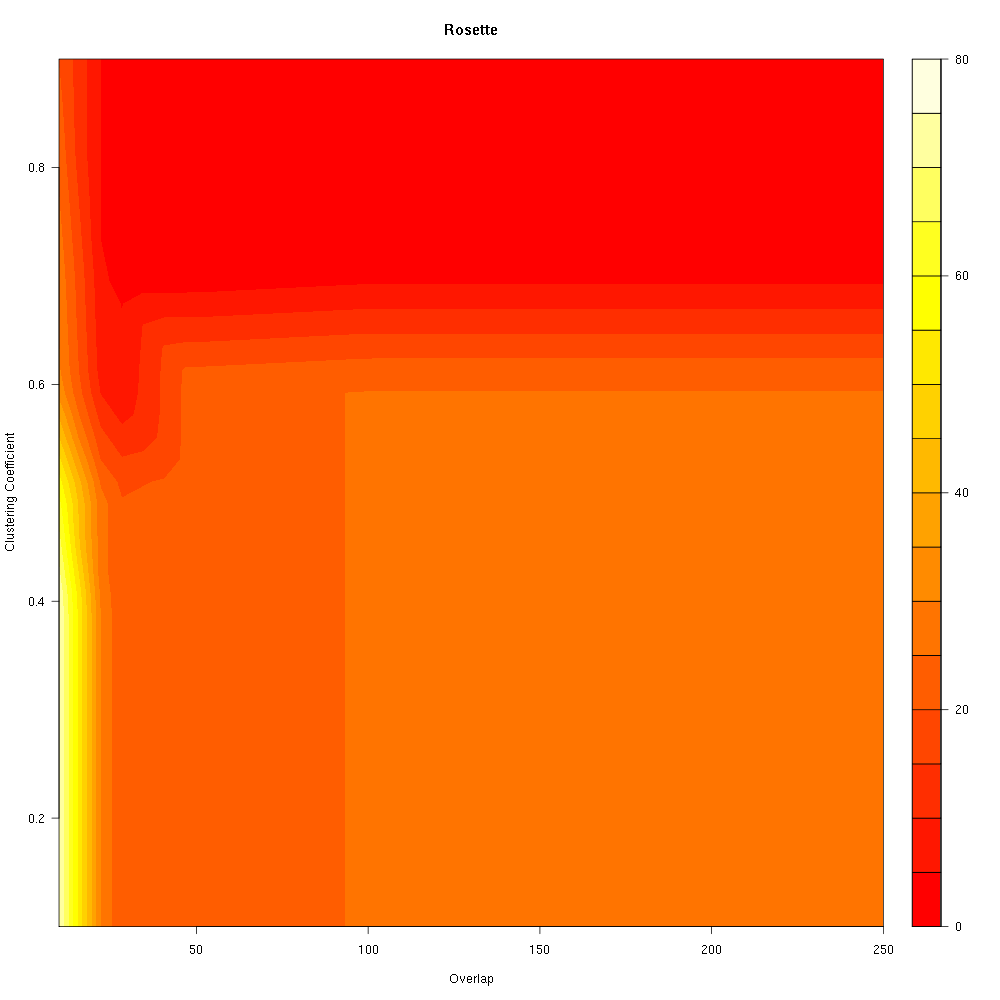

Supplement: Additional file 6 — Summary of parameter scans for sensitivity and specificity in mouse ES cells. [file 1471-2105-11-93-S6.PNG]

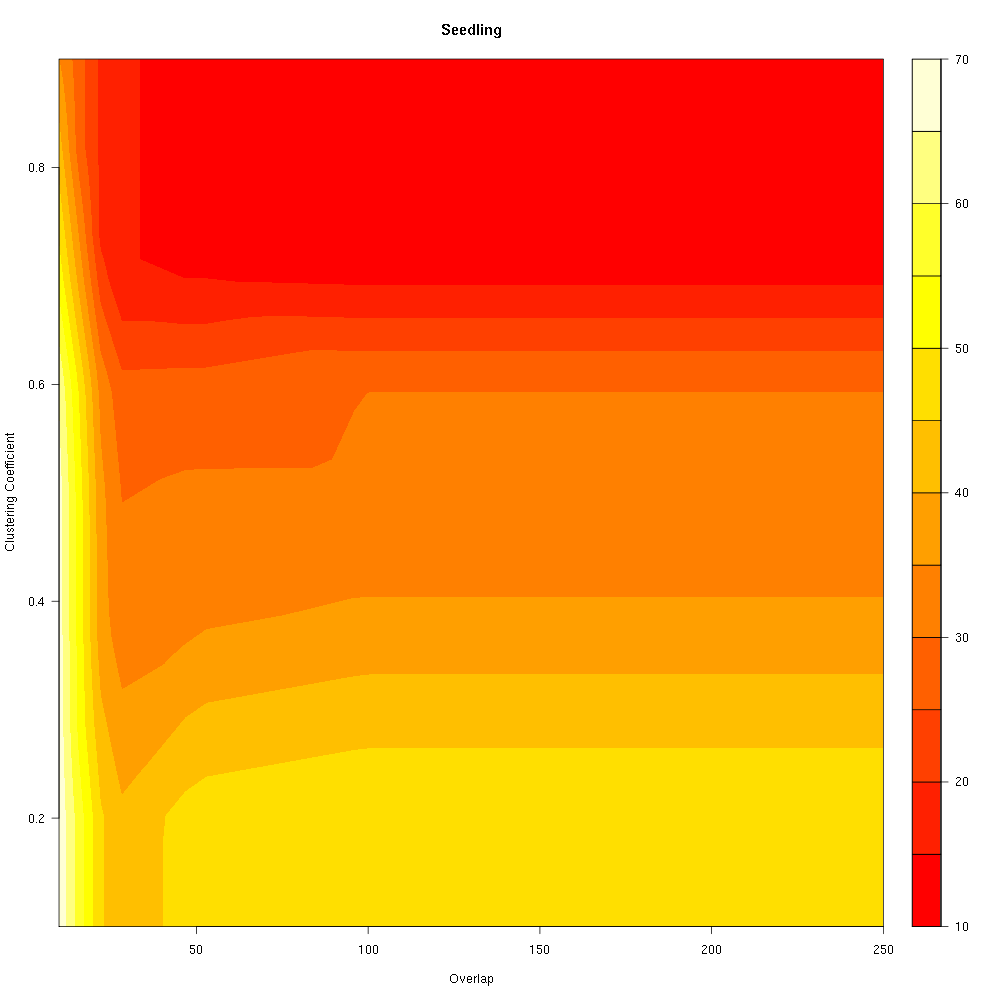

Supplement: Additional file 7 — Summary of parameter scans for sensitivity and specificity in Arabidopsis thaliana. [file 1471-2105-11-93-S7.PNG]
